# Dyshidrosiform Bullous Pemphigoid Triggered by COVID-19 Vaccination

**DOI:** 10.7759/cureus.26383

**Published:** 2022-06-28

**Authors:** Mohammed Shanshal

**Affiliations:** 1 Dermatology, Basildon University Hospital, Basildon, GBR

**Keywords:** dyshidrosiform bullous pemphigoid, bullous pemphigoid, covid 19, covid-19 vaccination skin side effects, covid-19 vaccination

## Abstract

Vaccination has made a substantial contribution to global health improvement throughout history. The coronavirus disease 2019 (COVID-19) vaccinations have provided us with a ray of hope for combatting the ongoing pandemic and saving lives. However, following COVID-19 vaccination, a wide spectrum of cutaneous adverse effects have been observed. We report a case of dyshidrosiform bullous pemphigoid, a rare clinical variant of bullous pemphigoid, following COVID-19 vaccination in an elderly female patient. The biopsy revealed subepidermal splitting with positive direct and indirect immunofluorescence studies.

## Introduction

Since the World Health Organization (WHO) declared it a global pandemic in March 2020, coronavirus disease 2019 (COVID-19), caused by severe acute respiratory syndrome coronavirus 2 (SARS-CoV-2), has affected millions of people worldwide. The massive impact of COVID-19 has led to the world's largest immunisation campaign. All approved COVID-19 vaccinations in the UK meet strict standards of safety, quality, and effectiveness [1]. While the COVID-19 vaccine's short-term adverse effects are similar to those of other vaccines, the vaccine's long-term side effects are still unknown, and rare systemic and cutaneous side effects continue to be reported throughout the world [2]. Bullous pemphigoid is a rare autoimmune blistering condition characterised by the development of sub-epithelial blisters. In most cases of bullous pemphigoid, no clear triggering cause has been identified. Possible predisposing factors have been linked to the development of bullous pemphigoid, including genetic susceptibility, concurrent medical conditions, certain medications, radiotherapy, and exposure to ultraviolet light [3].

## Case presentation

The patient was a 90-year-old woman who was admitted to the hospital on 30 March 2021 with a widespread itchy eczematous rash involving the trunk and extremities, and left leg swelling. The patient was on long-term warfarin, bisoprolol, amlodipine, bendroflumethiazide, and ursodeoxycholic acid for atrial fibrillation, hypertension, and primary biliary cirrhosis, respectively. Her blood pressure medications had been initiated more than 10 years ago, and her drug list had not changed recently. The patient’s family history was unremarkable, and she had never had a skin problem before.

The eruption had begun one week after her first COVID-19 vaccination (Pfizer-BioNTech) on 29 December 2020. Her general practitioner had tried hydrocortisone 1% cream, moisturiser, oral antihistamine, and permethrin 5% for suspected scabies. However, her skin condition persisted. After her second dose on 18 March 2021, the rash worsened considerably with severe itching.

On examination, there were widespread eczematous rash and excoriation marks involving the trunk and extremities. The face, palm, soles, and mucous membranes were spared. Left leg swelling was observed and the patient was investigated for possible deep vein thrombosis (DVT). Histology of the skin lesion showed features of the chronic eczematous process with mild perivascular chronic inflammation, and occasional neutrophils and eosinophils. The initial clinical impression was of a post-vaccination eczematous rash; the patient was discharged on oral prednisolone after ruling out DVT, and this vaccine reaction was reported through the UK Yellow Card Scheme. 

**Figure 1:**
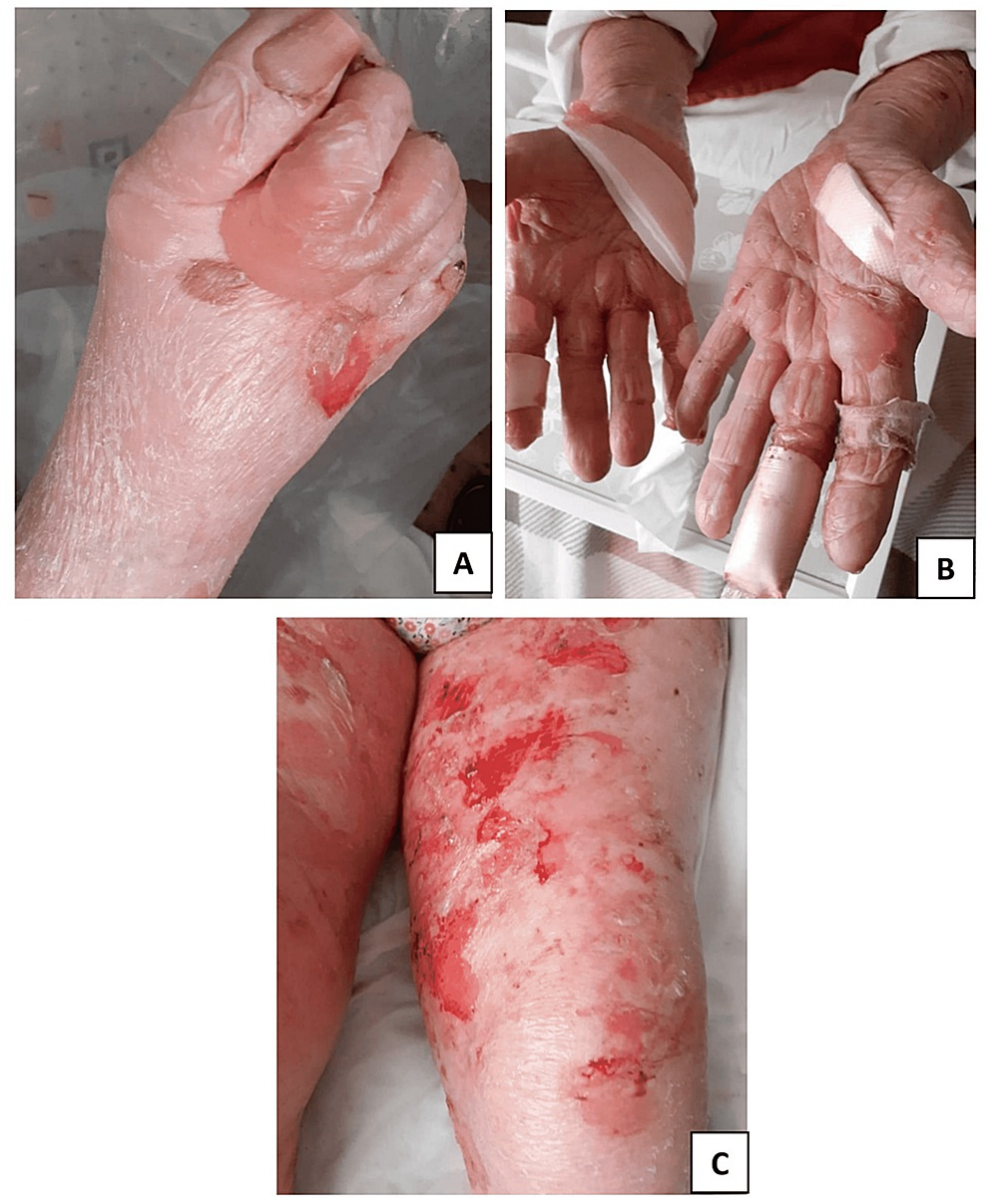
COVID-19-triggered dyshidrosiform bullous pemphigoid A and B: Tense bullae involving the palms and soles. C: Extensive erosions involving the extremities COVID-19: coronavirus disease 2019

On 16 May, the patient was re-admitted with severe itchy and painful large intact blisters involving the palms and soles (Figure 1).

**Figure 2:**
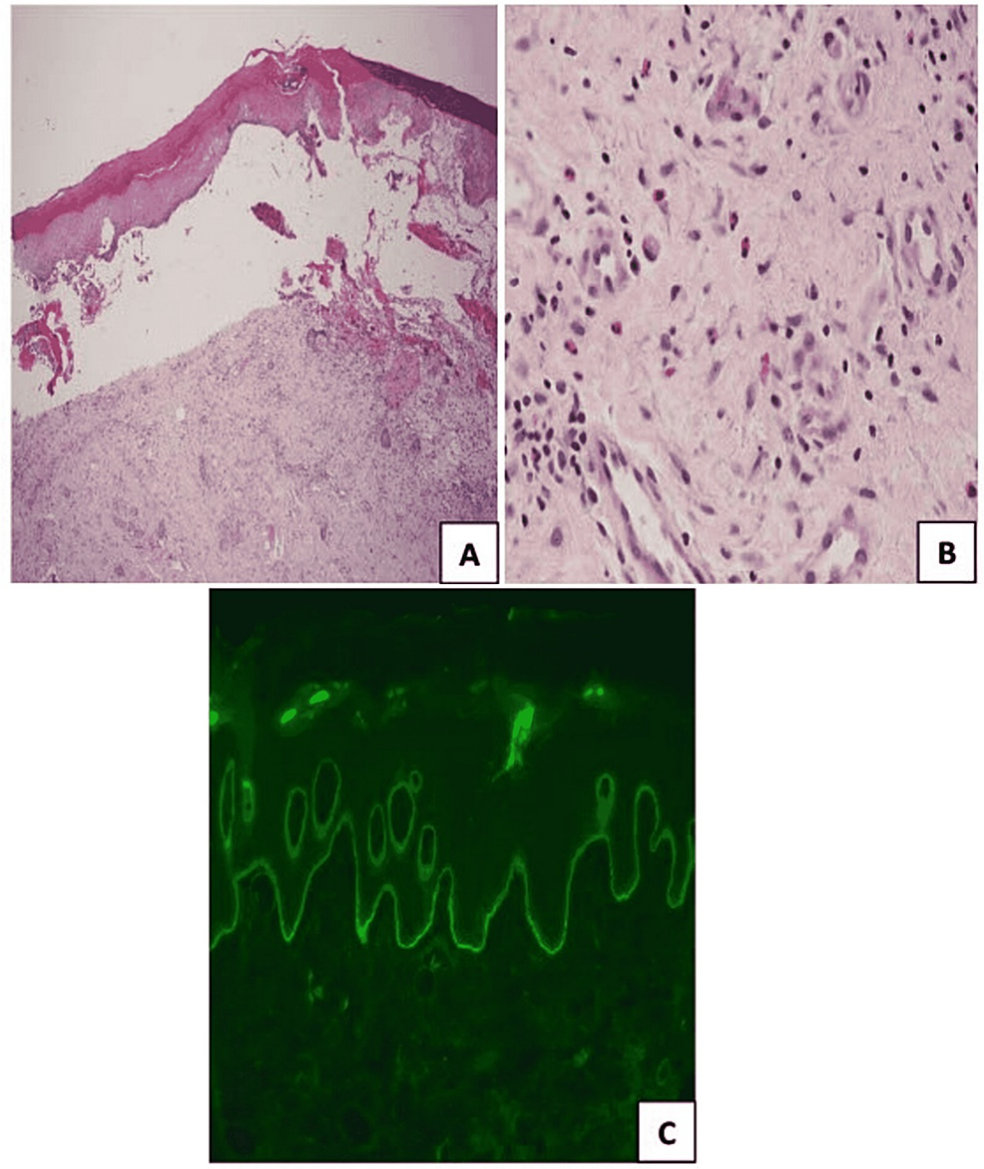
COVID-19-triggered dyshidrosiform bullous pemphigoid A and B: Hematoxylin and eosin (20x and 40x) showing subepidermal blistering with eosinophilic infiltrate. C: Perilesional direct immunofluorescence showing linear C3 at the basement membrane zone COVID-19: coronavirus disease 2019

Histological and immunofluorescence studies showed subepidermal splitting with moderate subjacent inflammation and linear deposition of C3 along the basement membrane zone (BMZ) respectively. Circulating anti-BMZ IgG autoantibodies were detected by the indirect immunofluorescence technique (Figure 2).

## Discussion

Bullous pemphigoid is an uncommon immunobullous disorder that typically presents in the elderly with generalised pruritic tense blisters on urticated or normal skin backgrounds. It is caused by acquired autoimmune IgG against two hemidesmosomal antigens (BpAg 180 and 230) in patients with underlying genetic predisposition [4]. Dyshidrosiform pemphigoid is a rare clinical variant of bullous pemphigoid where itchy blisters primarily involve the palms and soles and may mimic a vesicular eczema flare-up. This is sometimes followed by typical bullous lesions on other body sites [5,6].

Previous reports have suggested that bullous pemphigoid can be triggered by vaccination. Cases of bullous pemphigoid have been reported after the administration of tetanus [7], rabies [8], hepatitis B [9], influenza [10], meningococcus [11], and swine flu [12] vaccinations.

Pérez-López et al. have described a case of bullous pemphigoid induced by COMIRNATY® (Pfizer-BioNTech COVID-19 vaccine) three days after the vaccination [13]. COVID-19 vaccination has been reported to trigger flare-ups in three cases of bullous pemphigoid and two cases of pemphigus vulgaris in remission [14].

In our patient, the diagnosis of bullous pemphigoid was not made on the first admission due to the lack of an immunofluorescence test. Dyshidrosiform bullous pemphigoid can be reliably diagnosed based on the clinical presentation combined with histopathological and immunofluorescence results.

The temporal relationship between the onset of rash and the first dose, and the worsening of rash after the second dose support the hypothesis of a possible causative relationship between the administered vaccine and the appearance of bullous pemphigoid in our patient. Similar to drugs that are well-known triggers for bullous pemphigoid, vaccinations might modify the immune response or alter the antigenic properties of the BMZ, thereby inducing this immunobullous disorder [4].

## Conclusions

This report draws attention to the cutaneous adverse effects of COVID-19 vaccines and the importance to detect and report these complications as the global immunisation efforts continue. Bullous pemphigoid has been reported in the medical literature following COVID-19 vaccinations. We reported a case of dyshidrosiform bullous pemphigoid, an uncommon clinical variant that primarily affects the palms and soles following the administration of the COVID-19 Pfizer-BioNTech vaccine. This immunobullous condition could be caused by the vaccine affecting body immune responses or the antigenic features of the BMZ.
